# Supplementary material for: The interactome of intact mitochondria by cross-linking mass spectrometry provides evidence for coexisting respiratory supercomplexes
Source: Mol Cell Proteomics. 2017 Dec 8;17(2):216–32. doi: 10.1074/mcp.RA117.000470 (PMC5795388; doi:10.1074/mcp.RA117.000470)
Supplement: Supplemental Data [file supp_RA117.000470_133922_0_supp_23978_fzffwf.zip › spectra_annotation/mito_spectra_annotation/414-1-1-1-19-1.pdf]

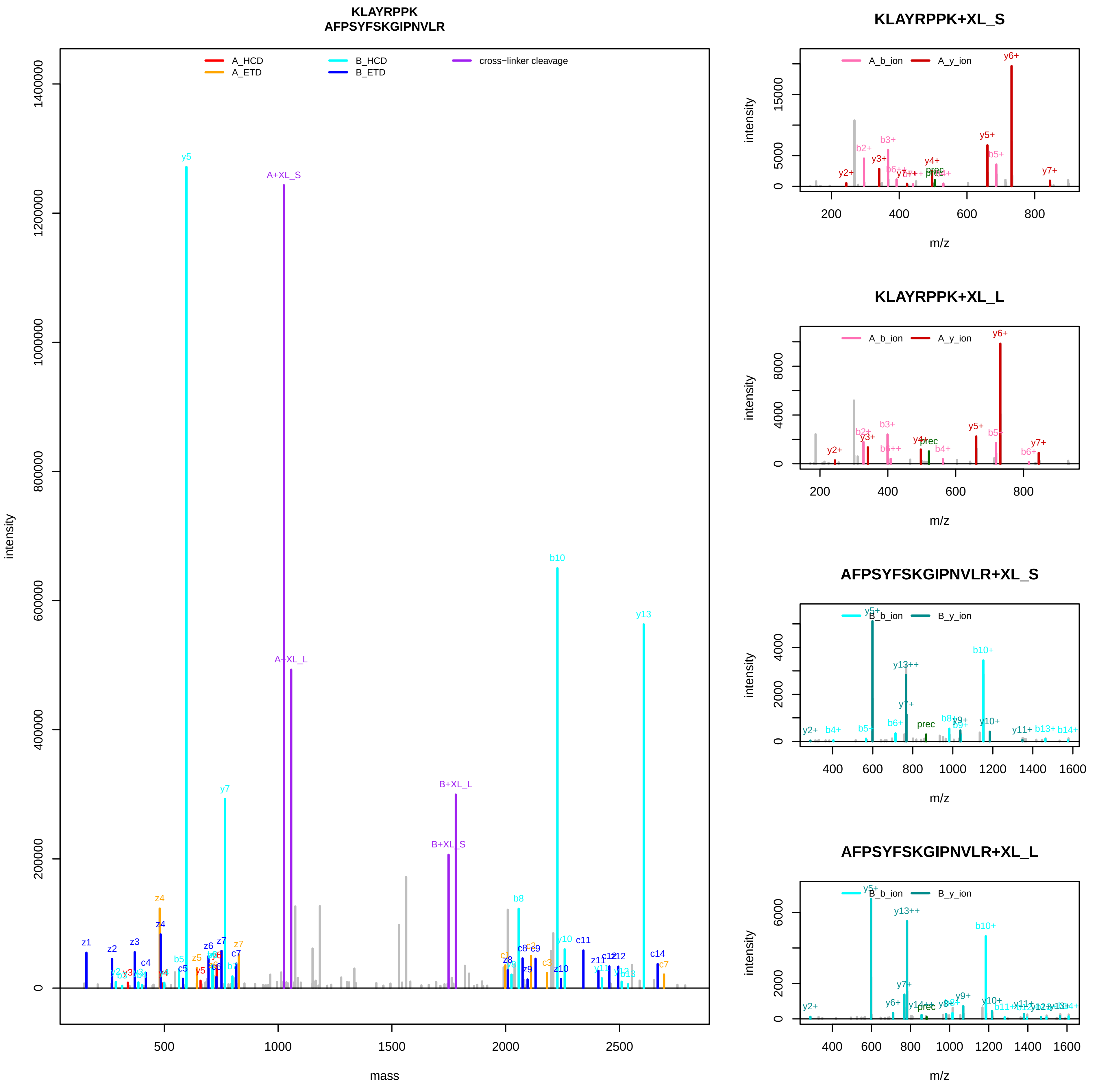
KLAYRPPK
AFPSYFSKGIPNVLR
A_HCD
A_ETD
B_HCD
B_ETD
cross−linker cleavage
y5
A+XL_S
A+XL_L
B+XL_L
B+XL_S
y7
b10
y13
b8
z4
intensity
mass
0
200000
400000
600000
800000
1000000
1200000
1400000
500
1000
1500
2000
2500
KLAYRPPK+XL_S
A_b_ion
A_y_ion
y6+
y5+
b5+
b3+
b2+
y3+
y2+
y4+
prec
y7+
intensity
m/z
0
5000
15000
200
400
600
800
KLAYRPPK+XL_L
A_b_ion
A_y_ion
y6+
y5+
b5+
b3+
y3+
y2+
y4+
prec
b4+
b6+
y7+
0
4000
8000
AFPSYFSKGIPNVLR+XL_S
B_b_ion
B_y_ion
y5+
y13++
b10+
y7+
b8+
y9+
y10+
prec
b6+
b5+
y2+
b4+
y11+
b13+
b14+
0
2000
4000
400
600
800
1000
1200
1400
1600
AFPSYFSKGIPNVLR+XL_L
B_b_ion
B_y_ion
y5+
y13++
b10+
y7+
y6+
y9+
y10+
y11+
y2+
prec
0
2000
6000
